# Supplementary material for: Heat adaptation of phage T7 under an extended genetic code
Source: Virus Evol. 2021 Dec 1;7(2):veab100. doi: 10.1093/ve/veab100 (PMC8923235; doi:10.1093/ve/veab100)
Supplement: veab100_Supp [file veab100_supp.zip › Sequencing_Analysis.zip!/Sequencing_Analysis/S1/evidence/JC_SIDE_1_59.html]

BRESEQ :: Evidence


| New junction evidence | | | | | | | | | | | |
| --- | --- | --- | --- | --- | --- | --- | --- | --- | --- | --- | --- |
|  | | seq id | position | reads (cov) | reads (cov) | score | skew | freq | annotation | gene | product |
| \* | ? | NC\_001604.1 | = 16604 | 1296 (0.310) | 133 (0.030) | 92/590 | 18.3 | 8.0% | intergenic (–/–) | *–/–* | –/– |
|  | ? | NC\_001604.1 | 17028 = | 1786 (0.430) |  |  |  |  | intergenic (–/–) | *–/–* | –/– |
| Rejected: Coverage evenness skew score above cutoff. | | | | | | | | | | | |
| Rejected: Frequency below/above cutoff threshold. | | | | | | | | | | | |

Only 100 of 1327 total aligned reads displayed.  

`GGGTACATGATGAAATCCAAGTAGGCTGCCGTACCGAAGAGATTGCTCAGGTGGTCATTGAGACCGCACAAGAAGCGATGCGCTGGGTTGGAGACCACTGGAACTTCCGGTGTCTTCTGGATACCGAAGGTAAGATGGGTCCTAATTGGGCGATTTGCCACTGATACAGGAGGCTACTCATGAACGAAAGACACTTAACAGGTGCTGCTTCTGAAATGCTAGTAGCCTACAAATTTACCAAAGCTGGGTACACTGTCTATTACCCTATGCTGACTCAGAGTAAAGAGGACTTGGTTGTATGTAAGGATGGTAAATTTAGTAAGGTTCAGGTTAAAACAGCCACAACGGTTCAAACCAACACAGGAGATGCCAAGCAGGTTAGGCTAGGTGGATGCGGTAGGTCCGAATATAAGGATGGAGACTTTGACATTCTTGCGGTTGTGGTTGACGAAGATGTGCTTATTTTCACATGGGACGAAGTAAAAGGTAAGACATCCATGTGTGTCGGCAAGAGAAACAAAGGCATAAAACTATAGGAGAAATTATTATGGCTATGACAAAGAAATTTAAAGTGTCCTTCGACGTTACCGCAAAGATGT  >  NC_001604.1/16304‑16902`  

                                                                                                                                                                                                                                                                                                            `|`                                                                                                                                                                                                                                                                                                            
`gggTACATGATGAAATCCAAGTAGGCTGCCGTACCGAAGAGATTGCTCAGGTGGTCATTGAGACCGCACAAGAAGCGATGCGCTGGGTTGGAGACCACTGGAACTTCCGGTGTCTTCTGGATACCGAAGGTAAGATGGGTCCTAATTGGGCGATTTGCCACTGATACAGGAGGCTACTCATGAACGAAAGACACTTAACAGGTGCTGCTTCTGAAATGCTAGTAGCCTACAAATTTACCAAAGCTGGGTACACTGTCTATTACCCTATGCTGACTAAGCGTAAAACGGACTTGGAtgtatg                                                                                                                                                                                                                                                                                                            >  1:400835/1‑301 (MQ=255)`  
 `ggTACATGATGAAATCCAAGTAGGCTGCCGTACCGAAGAGATTGCTCAGGTGGTCATTGAGACCGCACAAGAAGCGATGCGCTGGGTTGGAGAACACTGGAACTTCCGGTGTCTTCTGGATACCGAAGGTAAGATGGGTCCTAATTGGGCGATTTGCCACTGATACAGGAGGCTACTCATGAACGAAAGCCACTTAACAGGTGCTGCTTCTGAAATGCTAGTAGCCTACAACTTTACCAAAGCTGGGTACACTGTCTATTACCCTATGCTGACTCAGAGTAAAGCTGAATTGGTtgtatgt                                                                                                                                                                                                                                                                                                           >  1:670004/1‑301 (MQ=255)`  
 `ggTACATGATGAAATCCAAGTAGGCTGCCGTACCGAAGAGATTGCACAGGTGGTCATTGAGACCGCACAAGAAGCGATGCGCTGGGTTGGAGACCACTGGAACTTCCGGTGTCTTCTGGATACCGAAGGTAAGATGGGTCCTAATTGGGCGATTTGCCACTGATACAGGAGGCTACTCATGAACGAAAGACACTTAACAGGTGCTGCTTCTGAAATGCTAGTAGCCTACAAATTTACCAAAGCTGGGTACACTGTCTATTACCCTATGCTGACTCAGAGTAAAGAGGACTTGGTtgtatgg                                                                                                                                                                                                                                                                                                           <  1:569005/301‑2 (MQ=255)`  
  `gTACATGATGAAATCCAAGTAGGCTGCCGTACCGAAGAGATTGCTCAGGTGGCCATTGAGACCGCACAAGAAGCGATGCGCTGGGTTGGAGACCACTGGAACTTCCGGTGTCTTCTGGATACCGAAGGTAAGATGGGTCCTAATTGGGCGATTTGCCACTGATACAGGAGGCTACTCATGAACGAAAGACACTTAACAGGTGCTGCTTCTGAAATGCTAGTAGCCTACAAATTTACCAAAGCTGGGTACACTGTCTATTACCCTATGCTGACTCAGAGTAAAGAGGACTTGGTtgtatgga                                                                                                                                                                                                                                                                                                          >  1:111262/1‑299 (MQ=255)`  
      `atgatgAAATCCAAGTAGGCTGACGTACCGAAGAGATTGCTCAGGTGGTCATTGAGACCGCACAAGAAGCGATGCGCTGGGTTGGAGACCACTGGAACTTCCGGTGTCTTCTGGATACCGAAGGTAAGATGGGTCCTAATTGGGCGATTTGCCACTGATACAGGAGGCTACTCATGAACGAAAGACACTTAACAGGTGCTGCTTCTGAAATGCTAGTAGCCTACAAATTTACCAAAGCTGGGTACACTGTCTATTACCCTATGCTGACTCAGAGTAAAGAGGACTTGGTTGTATGTAAGGa                                                                                                                                                                                                                                                                                                      <  1:139701/301‑1 (MQ=255)`  
       `tgatgaAATCCAAGTAGGCTGCCGTACCGAAGAGATTGCTCAGGTGGTCATTGAGACCGCACAAGAAGCGATGCGCTGGGTTGGAGACCACTGGAACTTCCGGTGTCTTCTGGATACCGAAGGTAAGATGGGTCCTAATTGGGCGATTTGCCACTGATACAGGAGGCTACTCATGAACGAAAGACACTTAACAGGTGCTGCTTCTGAAATGCTAGTAGCCTACAAATTTACCAAAGCTGGGTACACTGTCTATTACCCTATGCTGACTCAGTGTAAAGAGGACTTGGTTGTATGTAAGGAt                                                                                                                                                                                                                                                                                                     <  1:147830/301‑1 (MQ=255)`  
             `aaTCCAAGTAGGCTGCCGTACCGAAGAGATTGCTCAGGTGGTCATTGAGACCGCACAAGAAGCGATGCGCTGGGTTGGAGACCACTGGAACTTCCGGTGTCTTCTGGATACCGAAGGTAAGATGGGTCCTAATTGGGCGATTTGCCACTGATACAGGAGGCTACTCATGAACGAAAGACAATTAACAGGTGCTGCTTCTGAAATGCTAGTAGCCTACAAATTTACCAAAGCTGGGTGCACTGTCTATTACCATATGCTGACTCAGAGTAAAGAGGACGTGGTTGTATGTAAGGATgtctct                                                                                                                                                                                                                                                                                               >  1:37734/1‑296 (MQ=255)`  
                 `cAAGTAGGCTGCCGTACCGAAGAGATTGCTCAGGTGGTCATTGAGACCGCACAAGAAGCGATGCGCTGGGTTGGAGACCACTGGAACTTCCGGTGTCTTCTGGATACCGAAGGTAAGATGGGTCCTAATTGGGCGATTTGCCACTGATACAGGAGGCTACTCATGAACGAAAGACACTTAACAGGTGCTGCTTCTGAAATGCTAGTAGCCTACAAATTTACCAAAGCTGGGTACACTGTCTATTACCCTATGCTGACTCAGAGTAAAGAGGACTTGGTTGTATGTAAGGATGGTAAATTTa                                                                                                                                                                                                                                                                                           >  1:489690/1‑301 (MQ=255)`  
                     `tAGGCTGCCGTACCGAAGAGATTGCTCAGGTGGTCATTGAGACCGCACAAGAAGCGATGCGCTGGGTTGGAGACCACTGGAACTTCCGGTGTCTTCTGGATACCGAAGGTAAGATGGGTCCTAATTGGGCGATTTGCCACTGATACAGGAGGCTACTCATGAACGAAAGACACTTAACAGGTGCTGCTTCTGAAATGCTAGTAGCCTACAAATTTACCAAAGCTGGGTACACTGTCTATTACCCTATGCTGACTCAGAGTAAAGAGGACTTGGTTGTATGTAAGGATGGTAAATTTAGTaa                                                                                                                                                                                                                                                                                       <  1:24677/301‑1 (MQ=255)`  
                     `tAGGCTGCCGTACCGAAGAGATTGCTCAGGTGGTCATTGAGACCGCACAAGAAGCGATGCGCTGGGTTGGAGACCACTGGAACTTCCGGTGTCTTCTGGATACCGAAGGTAAGATGGGTCCTAATTGGGCGATTTGCCACTGATACAGGAGGCTACTCATGAACGAAAGACACTTAACAGGTGCTGCTTCTGAAATGCTAGTAGCCTACAAATTTACCAAAGCTGGGTACACTGTCTATTACCCTATGCTGACTCAGAGTAAAGAGGACTTGGTTGTATGTAAGGATGGTAAATTTAGTaa                                                                                                                                                                                                                                                                                       <  1:322437/301‑1 (MQ=255)`  
                     `agatgtgtataagagacaggaTTGCTCAGGTGGTCATTGAGACCGCACAAGAAGCGATGCGCTGGGTTGGAGACCACTGGAACTTCCGGTGTCTTCTGGATACCGAAGGTAAGATGGGTCCTAATTGGGAGATTTGCAACTGATAAAGGAGGCTACTCATGAACGAAAGACACTTAACAGGTGCTGCTTCTGAAATGCTAGTAGACTACAAATTTACCAAAGCTGGGTACACTGTCTATTACCCTATGCTGACTCAGAGTAAAGAGGAATTGGTTGTATGTAAGGATGGTAAATTTAGTaa                                                                                                                                                                                                                                                                                       <  1:224985/282‑1 (MQ=255)`  
                      `aGGCTGCCGTACCGAAGAGATTGCTCAGGTGGTCATTGAGACCGCACAAGAAGCGATGCGCTGGGTTGGAGACCACTGGAACTTCCGGTGTATTCTGGATACCGAAGGTAAGATGGGTCCTAATTGGGCGCTTTGCCACTGATACAGGAGGCTACTCATGAACGAAAGACACTTAACAGGTGCTGCTTCTGAAATGCTAGTAGCCTACAAATTTACCAAAGCTGGGTACACTGTCTATTACCCTATGCTGACTCAGAGTACAGAGGACTTGGTTGTATGTAAGGATGGAAAATTTAGTAAg                                                                                                                                                                                                                                                                                      >  1:661453/1‑301 (MQ=255)`  
                       `ggCTGCCGTACCGAAGAGATTGCTCAGGTGGTCATTGAGACCGCACAAGAAGCGATGCGCTGGGTTGGAGACCACTGGAACTTCCGGTGTCTTCTGGATACCGAAGGTAAGATGGGTCCTAATTGGGCGATTTGCCACTGATACAGGAGGCTACTCATGAACGAAAGACACTTAACAGGTGCTGCTTCTGAAATGCTAGCAGCCTACAAATTTACCAAAGCTGGGTACACTGTCTATTACCCTATGCTGACTCAGAGTAAAGAGGACTTGGTTGTATGTAAGGactgtctcttatacacat                                                                                                                                                                                                                                                                                     >  1:117966/1‑284 (MQ=255)`  
                           `gCCGTACCGAAGAGATTGCTCAGGTGGTCATTGAGACCGCACAAGAAGCGATGCGCTGGGTTGGAGACCACTGGAACTTCCGGTGTCTTCTGGATACCGAAGGTAAGATGGGTCCTAATTGGGCGATTTGCCAATGATACAGGAGGCTACTCATGAACGAAAGACACTTAACAGGTGCTGTTTCTGAAATGCTAGTAGACTACAAATTTACCAAAGCTGGGTACACTGTCTATTACACTATGCTGACTCAGAGTAAAGAGGACTTGGTtgtatgtctggctcttatacacatctccgagcc                                                                                                                                                                                                                                                                                 >  1:197580/1‑275 (MQ=255)`  
                              `gTACCGAAGAGATTGCTCAGGTGGTCATTGAGACCGCACAAGAAGCGATGCGCTGGGTTGGAGACCACTGGAACTTCCGGTGTCTTCTGGATACCGAAGGTAAGATGGGTCCTAATTGGGCGATTTGCCACTGATACAGGAGGCTACTCATGAACGAAAGACACTTAACAGGTGCTGCTTCTGAAATGCTAGTAGCCTACAAATTTACCAAAGCTGGGTACACTGTCTATTACCCTATGCTGACTCAGAGTAAAGAGGACTTGGTTGTATGTAAGGactggctcttatacaaatctccgag                                                                                                                                                                                                                                                                              >  1:121437/1‑277 (MQ=255)`  
                                `aCCGAAGAGATTGCTCAGGTGGTCATTGAGACCGCACAAGAAGCGATGCGCTGGGTTGGAGACCACTGGAACTTCCGGTGTCTTCTGGATACCGAAGGTAAGATGGGTCCTAATTGGGCGATTTGCCACTGATACAGGAGGCTACTCATGAACGAAAGACACTTAACAGGTGCTGCTTCTGAAATGCTAGTAGCCTACAAATTTACCAAAGCTGGGTACACTGTCTATTACCCTATGCTGACTCAGAGTAAAGAGGACTTGGTTGTATGTAAGGATGGTAAATTTAGTAAGGTTCAGGTTa                                                                                                                                                                                                                                                                            >  1:499162/1‑301 (MQ=255)`  
                                    `aaGTGATTGCTAAGGTTGTCATTGAGACCGCACAAGAAGCGATGCGCTGGGTTGGAGACCACTGGAACTTCCGGTGTCTTCTTGATACCGAAGGTAAGATGGGTCCTAATTGGGCGATTTGCCACTGATACAGGAGGCTACTCATGAACGAAAGACACTTAACAGGTGCTGCTTCTGAAATGCTAGTATCCTACAAATTTACCAAAGCTGGGTACACTGTCTATTACCCTATGCTGACTCAGAGTAAAGAGGACTTGGTTGTATGTAAGGATGGTAAATTTAGTAAGGTTCAGGTTAAAAc                                                                                                                                                                                                                                                                        <  1:530043/301‑1 (MQ=255)`  
                                    `aaGAGATTGCTCAGGTGGTCATTGAGACCGCACAAGAAGCGATGCGCTGGGTTGGAGACCACTGGTACTTCCGGTGTCTTCTGGATACCGAAGGTAAGATGGGTCCTAATGGGGCGATTTGCCACTGATACAGGAGGCTACTCATGAACGAAAGACACTTAACAGGTGCTGCTTCTGAAATGCTAGTAGCCTACAAATTTACCAAAGCTGGGTACACTGTCTATTACCCTATGCTGACTCAGAGTAAAGAGGACTTGGTTGTATGTAAGGATGGTAAATTTAGTAAGGTTCAGGTTAAAAc                                                                                                                                                                                                                                                                        <  1:218167/301‑1 (MQ=255)`  
                                                 `ggtggtCATTGAGACCGCACAAGAAGCGATGCGCTGGGTTGGAGACCACTGGAACTTCCGGTGTCTTCTGGATACCGAAGGTAAGATGGGTCCTAATTGGGCGATTTGCCACTGATACAGGAGGCTAATCATGAACGAAAGACACTTAACAGGTGCTGCTTCTGAAATGCTAGTAGCCTCCAAATTTACCAAAGCTGGGTACACTGTCTCTTTCCCTATTCTGACTCAGAGTAAAGAGGACTTGGTTGTATTTAAGGATTGTAGATTTAGTAAGGTTCAGGTTAAAACAGCCACAACGGtt                                                                                                                                                                                                                                                           >  1:126446/1‑301 (MQ=255)`  
                                                       `taTTGAGACCGCAGAAGAAGCGATGCGCTGGGTTGGAGACCACTGGAACGTACGGTGTCTTCTGGATACCGAAGGTAAGATGGGTCCTAATTGTGCGATTTGCCACTGATACAGGAGGCTACTCATGAACGAAAGACACTTAACAGGTGCTGCTTCTGAAATGCTAGTAGACTACAAATTTACCAAAGCTGGGTACACTGTCTATTACCCTATGCTGACTCAGAGTAAAGAGGACTTGGTTGTATGTAAGGATGGTAAATTTAGTAAGGTTCAGGTTAAAACAGCCACAACGGTTCAAAcc                                                                                                                                                                                                                                                     <  1:362099/300‑1 (MQ=255)`  
                                                       `cATTGAGACCGCACAAGAAGCGATGCGCTGGGTTGGAGACCACTGGAACTTCCGGTGTCTTCTGGATACCGAAGGTAAGATGGGTCCTAATTGGGCGATTTGCCACTGATACAGGAGGCTACTCATGAACGAAAGACACTTAACAGGTGCTGCTTCTGAAATGCTAGTAGCCTACAAATTTACCAAAGCTGGGTACACTGTCTATTACCCTATGCTGACTCAGAGTAAAGAGGACTTTGTTGTATGTAAGGATGGTAAATTTAGTAAGGTTCAGGTTAAAAcctgtctcttatacacatct                                                                                                                                                                                                                                                     >  1:413710/1‑282 (MQ=255)`  
                                                            `tataagagacagaagCGATGCGCTGGGTTGGAGACCACTGGAACTTCCGGTGTCTTCTGGATACCGAAGGTAAGATGGGTCCTAATTGGGCGATTTGCCACTGATACAGGAGGCTACTCATGAACGAAAGACACTTAACAGGTGCTGCTTCTGAAATGCTAGTAGCCTACAAATTTACCAGAGCTGGGTACACTGTCTATTACCCTATGCTGACTCAGAGTAAAGAGGACTTGGTTGTATGTAAGGATGGTAAATTTAGTAAGGTTCAGGTTAAAACAGCCACAACGGTTCAAACCAacac                                                                                                                                                                                                                                                <  1:542507/291‑1 (MQ=255)`  
                                                                 `gaacaAGAAGCGATGCGCTGGTGTGGAGACCACTGGAACTTCCGGTGTCTTCTGGATACCGAAGGTAAGATGGGTCCTAATTGGGCGATTTGCCACTGATACAGGAGGCTACTCATGAACGAAAGACACTTAACAGGTGCTGCTTCTGAAATGCTAGTAGCCTACAAATTTACCAAAGCTGGGTACACTGTCTATTACCCTATGCTGACTCAGAGTAAAGAGGAATTGGTTGTATGTAAGGATGGTAAATTTAGTAAGGTTCAGGTTAAAACAGCCACAACGGTTCAAACCAACACAGgag                                                                                                                                                                                                                                           <  1:484787/299‑1 (MQ=255)`  
                                                                 `gCACAAGAAGCGATGCGCTGGGTTGGAGACCACTGGAACTTCCGGTGTCTTCTGGATACCGAAGGTAAGATGGGTCCTAATTGGGCGATTTGCCACTGATACAGGAGGCTACTCATGAACGAAAGACACTTAACAGGTGCTGCTTCTGAAATGCTAGTAGCCTACAAATTTACCAAAGCTGGGTACACTGTCTATTACCCTATGCTGACTCAGAGTAAAGAGGACTTGGTTGTATGTAAGGATGGTAAATTTAGTAAGGTTCAGGTTAAAACAGCCACAACGGTTCAAACCAACACAGgag                                                                                                                                                                                                                                           <  1:515826/301‑1 (MQ=255)`  
                                                                      `agaagCGATGCGGTGGGTTGGAGACCACTGGAACTTCAGGTGTCTGCTGGATACCGAAGGGAAGATGGGTCCTAATTGGGCGAGTGGCTACTGAGACAGGAGGCTACTCATGAACGAAAGACACTTAACATGTGCTGCTTCTGAAATGCTAGTAGCCTACAAATTTACCAAAGCTGGGTACACTGTCTATTACCCTATGCTGACTCAGAGTAAAGAGGACTTGGTTGTATGTAAGGATGGTAAATTTAGTAAGGTTCAGGTTAAAACAGCCACAACGGTTCAAACCAACACAGGAGATGcc                                                                                                                                                                                                                                      <  1:426790/301‑1 (MQ=255)`  
                                                                      `agaagCGATGCGCTGGGTTGGAGACCACTGGAACTTCCGGTGTCTTCTGGATACCGAAGGTAAGATGGGTCCTAATTGGGCGATTTGCCACTGATACAGGAGGCTACTCATGAACGAAAGACACTTAACAGGTGCTGCTTCTGAAATGCTAGTAGCCTACAAATTTACCAAAGCTGGGTACAATGTCTATTACCCTATGCTGACTCAGAGTAAAGAGGACTTGGTTGTATGTAAGGCTGGTAAATTTAGTATGGTTCAGGTTAAAACAGCCACAACGGTTCAAACCAACACAGGAGATGcc                                                                                                                                                                                                                                      >  1:480022/1‑301 (MQ=255)`  
                                                                               `gcgcTGGGTTGGAGACCACTGGAACTTCCGGTGTCTTCTGGATACCGAAGGTAAGATGGGTCCTAATTGGGCGATTTGCCACTGATACAGGAGGCTACTCATGAACGAAAGACACTTAACAGGTGCTGCTTCTGAAATTCTAGTAGCCTACAAATTTACCAAAGCTGGGTACACTGTCTATTACCTTCTGCTGACTCAGAGTAAAGAGGACTTGGTTGTATGTAAGGATGGTAAATTTAGTAAGGTTCAGGTTAAAACAGCCACAACGGTTAAAACCAACACAGGAGCTgtctcttataca                                                                                                                                                                                                                             >  1:157609/1‑290 (MQ=255)`  
                                                                                    `gggTTGGAGACCACTGGAACTTCCGGTGTCTTCTGGATACCGAAGGTAAGATGGGTCCTAATTGGGCGATTTGCCACTGATACAGGAGGCTACTCATGAACGAAAGACACTTAACAGGTGCTGCTTCTGAAATGCTAGTAGCCTACAAATTTACCAAAGCTGGGTACACTGTCTATTACCCTATGCTGACTCAGAGTAAAGAGGACTTGGTTGTATGTAAGGATGGTAAATTTAGTAAGGTTCAGGTTAAAACAGCCACAACGGTTCAAACCAACACAGGAGCTgtctcttatccacatct                                                                                                                                                                                                                        >  1:238411/1‑285 (MQ=255)`  
                                                                                         `ggAGACCACTGGAACTTCCGGTGTCTTCTGGATACCGAAGGTAAGATGGGTCCTAATTGGGCGATTTGCCACTGATACAGGAGGCTACTCATGAACGAAAGACACTTAACAGGTGCTGCTTCTGAAATGCTAGTAGCCTACAAATTTACCAAAGCTGGGTACACTGTCTATTACCCTATGCTGACTCAGAGTAAAGAGGACTTGGTTGTATGTAAGGATGGTAAATTTAGTAAGGTTCAGGTTAAAACAGCCACAACGGTTCAAACCAACACAGGAGATGCCAAGCAGGTTAGGCTAGGTg                                                                                                                                                                                                                   <  1:616077/301‑1 (MQ=255)`  
                                                                                         `ggAGACCACTGGAACTTCCGGTGTCTTCTGGATACCGAAGGTAAGATGGGTCCTAATTGGGCGATTTGCCACTGATACAGGAGGCTACTCATGAACGAAAGACACTTAACAGGTGCTGCTTCTGAAATGCTAGTAGCCTACAAATTTACCAAAGCTGGGTACACTGTCTATTACCCTATGCTGACTCAGAGTAAAGAGGACTTGGTTGTATGTAAGGATGGTAAATTTAGTAAGGTTCAGGTTAAAACAGCCACAACGGTTCAAACCAACACAGGAGATGCCAAGCAGGTTAGGCTAGGTg                                                                                                                                                                                                                   <  1:636927/301‑1 (MQ=255)`  
                                                                                            `gaCCACTGGAACTTCCGGTGTCTTCTGGATACCGAAGGTAAGATGGGTCCTAATTGGGCGATTTGCCACTGATACAGGAGGCTACTCATGAACGAAAGACACTTAACAGGTGCTGCTTCTGAAATGCTAGTAGCCTACAAATTTACCAAAGCTGGGTACACTGTCTATTACCCTATGCTGACTCAGAGTAAAGATGACTTGGTTGTATGTAAGGATGGTAAATTTAGTAAGGTTCAGGTTAAAACAGCCACAACGGTTCAAACCAACACAGGAGATGCCAAGCAGGTTAGGCtctgtctct                                                                                                                                                                                                                >  1:345043/1‑293 (MQ=255)`  
                                                                                                             `gtgtCTTCTGGATACCGAAGGTAAGATGGGTCCTAATTGGGCGATTTGCCACTGATACAGGAGGCTACTCATGAACGAAAGACACTTAACAGGTGCTGCTTCTGAAATGCTAGTAGCCTACAAATTTACCAAAGCTGGGTACACTGTCTATTACCCTATGCTGACTCAGAGTAAAGAGGACTTGGTTGTATGTAAGGATGGTAAATTTAGTAAGGTTCAGGTTAAAACAGCCACAACGGTTCAAACCAACACAGGAGATGCCAAGCAGGTTAGGCTAGGTGGATGCGGTAGGTCCGAatat                                                                                                                                                                                               <  1:295116/301‑1 (MQ=255)`  
                                                                                                                      `ggATACCGAAGGTAAGATGGGTCCTAATTGGGCGATTTGCCACTGATACAGGAGGCTACTCATGAACGAAAGACACTTAACAGGTGCTGCTTCTGAAATGCTAGTAGCCTACAAATTTACCAAAGCTGGGTACACTGTCTATTACCCTATGCTGACTCAGAGTAAAGAGGACTTGGTTGTATGTAAGGATGGTAAATTTAGTAAGGTTCAGGTTAAAACAGCCACAACGGTTCAAACCAACACAGGAGATGCCAAGCAGGTTAGGCTAGGTGGATGCGGTAGGTCCGAATATAAGGATGga                                                                                                                                                                                      >  1:253243/1‑301 (MQ=255)`  
                                                                                                                       `gATACCGAAGGTAAGATGGGTCCTAATTGGGCGATTTGCCACTGATACAGGAGGCTACTCATGAACGAAAGACACTTAACAGGTGCTGCTTCTGAAATGCTAGTAGCCTACAAATTTACCAAAGCTGGGTACACTGTCTATTACCCTATGCTGACTCAGAGTAAAGAGGACTTGGTTGTATGTAAGGATGGTAAATTTAGTAAGGTTCAGGTTAAAACAGCCACAACGGTTCAAACCAACACAGGAGATGCCAAGCAGGTTAGGCTAGGTGGATGCGGTAGGTCCGAATATAAGGATGgag                                                                                                                                                                                     <  1:78833/301‑1 (MQ=255)`  
                                                                                                                                `ggTAAGATGGGTCCTAATTGGGCGATTTGCCACTGATACAGGAGGCTACTCATGAACGAAAGACACTTAACAGGTGCTGCTTCTGAAATGCTAGTAGCCTACAAATTTACCAAAGCTGGGTACACTGTCTATTACCCTATGCTGACTCAGAGTAAAGAGGACTTGGTTGTATGTAAGGATGGTAAATTTAGTAAGGTTCAGGTTAAAACAGCCACAACGGTTCAAACCAACACAGGAGATGCCAAGCAGGTTAGGCTAGGTGGATGCGGTAGGTCCGAATATAAGGactgtctcttataca                                                                                                                                                                            >  1:62289/1‑287 (MQ=255)`  
                                                                                                                                `ggTAAGATGGGTCCTAATTGGGCGATTTGCCACTGATACAGGAGGCTACTCATGAACGAAAGACACTTAACAGGTGCTGCTTCTGAAATGCTAGTAGCCTACAAATTTACCAAAGCTGGGTACACTGTCTATTACCCTATGCTGACTCAGAGTAAAGAGGACTTGGTTGTATGTAAGGATGGTAAATTTAGTAAGGTTCAGGTTAAAACAGCCACAACGGTTCAAACCAACACAGGAGATGCCAAGCAGGTTAGGCTAGGTGGATGCGGTAGGTCCGAATATAAGGATGGAGACTTTGACa                                                                                                                                                                            >  1:178410/1‑301 (MQ=255)`  
                                                                                                                                `ggTAAGATGGGTCCTAATTGGGCGATTTGCCACTGATACAGGAGGCTACTCATGAACGAAAGACACTTAACAGGTGCTGCTTCTGAAATGCTAGTAGCCTACAAATTTACCAAAGCTGGGTACACTGTCTATTACCCTATGATGACTCAGAGTAAAGAGGACTTGGTTGTATGTAAGGATGGTAAATTTAGTAAGGTTCAGGTTAAAACAGCCACAACGGTTCAAACCAACACAGGAGATGCCAAGCAGGTTAGGCTAGGTGGATGCGGTAGGTCCGAATATAAGGATGGAGACTTTGACa                                                                                                                                                                            >  1:469309/1‑301 (MQ=255)`  
                                                                                                                                                  `tGGGCGATTTGCCACTGATACAGGAGGCTACTCATGAACGAAAGACACTTAACAGGTGCTGCTTCTGAAATGCTAGTAGCCTACAAATTTACCAAAGCTGGGTACACTGTCTATTACCCTATGCTGACTCAGAGTAAAGAGGACTTGGTTGTATGTAAGGATGGTAAATTTAGTAAGGTTCAGGTTAAAACAGCCACAACGGTTCAAACCAACACAGGAGATGCCAAGCAGGTTAGGCTAGGTGGATGCGGTAGGTCCGAATATAAGGATGGAGACTTTGACATTCTTGCGGTTGTGGTTg                                                                                                                                                          <  1:503204/301‑1 (MQ=255)`  
                                                                                                                                                     `agagacagCCACTGATACAGGAGGCTACTCATGAACGAAAGACACTTAACAGGTGCTGCTTCTGAAATGCTAGTAGCCTACAAATTTACCAAAGCTGGGTACACTGTCTATTACCCTATGCTGACTCAGAGTAAAGAGGACTTGGTTGTATGTAAGGATGGTAAATTTAGTAAGGTTCAGGTTAAAACAGCCACAACGGTTCAAACCAACACAGGAGATGCCAAGCAGGTTAGGCTAGGTGGATGCGGTAGGTCCGAATATAAGGATGGAGACTTTGACATTCTTGCGGTTGTGGTTGACg                                                                                                                                                       <  1:83468/294‑1 (MQ=255)`  
                                                                                                                                                      `cGATTTGCCACTGATACAGGAGGCTACTCATGAACGAAAGACACTTAACAGGTGCTGCTTCTGAAATGCTAGTAGCCTACAAATTTACCAAAGCTGGGTACACTGTCTATTACCCTATGCTGACTCAGAGTAAAGAGGACTTGGTTGTATGTAAGGATGGTAAATTTAGTAAGGTTCAGGTTAAAACAGCCACAACGGTTCAAACCAACACAGGAGATGCCAAGCAGGTTAGGCTAGGTGGATGCGGTAGGTCCGAATATAAGGATGGAGACTTTGACATTCTTGCGGTTGTGGTTGACGa                                                                                                                                                      <  1:275955/301‑1 (MQ=255)`  
                                                                                                                                                                `cTGATACAGGATGCTACTCATGAACGAAAGACACTTAACAGGTGCTGCTTCTGAAATGCTAGTATCCTACAAATTTACCAAAGCTGGGTACACTGTCTATTACCCTATGCTGACTCAGAGTAAAGAGGACTTGGTTGTATGTAAGGATGGTAAATTTAGTAAGGTTCAGGTTAAAACAGCCACAACGGTTCAAACCAACACAGGAGATGCCAAGCAGGTTAGGCTAGGTGGATGCGGTAGGTCCGAATATAAGGATGGAGACTTTGACATTCTTGCGGTTGTGGTTGACGAAGATGTGCtt                                                                                                                                            >  1:397788/1‑301 (MQ=255)`  
                                                                                                                                                                 `tGATACAGGAGGCTACTCATGAACGAAAGACACTTAACAGGTGCTGCTTCTGAAATGCTAGTAGCCTACAAATTTACCAAAGCTGGGTACACTGTCTATTACCCTATGCTGACTCAGAGTAAAGAGGACTTGGTTGTATGTAAGGATGGTAAATTTAGTAAGGTTCAGGTTAAAACAGCCACAACGGTTCAAACCAACACAGGAGATGCCAAGCAGGTTAGGCTAGGTGGATGCGGTAGGTCCGAATATAAGGATGGAGACTTTGACATTCTTGCGGTTGTGGTTGACGAGGATGCGCttt                                                                                                                                           >  1:508548/1‑300 (MQ=255)`  
                                                                                                                                                                   `aTACAGGAGGCTACTCATGAACGAAAGACACTTAACAGGTGCTGCTTCTGAAATGCTAGTAGCCTACAAATTTACCAAAGCTGGGTACACTGTCTATTACCCTATGCTGACTCAGAGTAAAGAGGACTTGGTTGTATGTAAGGATGGTAAATTTAGTAAGGTTCAGGTTAAAACAGCCACAACGGTTCAAACCAACACAGGAGATGCCAAGCAGGTTAGGCTAGGTGGATGCGGTAGGTCCGAATATAAGGATGGAGACTTTGACATTCTTGCGGTTGTGGTTGACGAAGATGTGCTTAtt                                                                                                                                         >  1:228809/1‑301 (MQ=255)`  
                                                                                                                                                                          `gtgtataagagacaggAAAGACACTTAACAGGTGCTGCTTCTGAAATGCTAGTAGCCTACAAATTGACCAAAGATGGGTACACTGTCTATTACACTATGCTGAATCCGAGTAAAGAGGACTTGGTTGTATGTAAGGATGGTAAATGTAGTAAGGTTCAGGGTAAAACAGCCACAACGGTTCAAACCAACACAGGAGATGCCAAGCAGGTTAGGCTAGGTGGATGCGGTAGGCTCGAATATAAGGATGGAGACTTTGACATTCTTGCGGTTGTGGTTGACGAAGATGTGCTTATTTTCACAt                                                                                                                                  <  1:309228/286‑1 (MQ=255)`  
                                                                                                                                                                          `agacagcTCATGAACGAAAGACACTTAACAGGTGCTGCTTCTGAAATGCTAGTAGCCTACAAATTTACCAAAGCTGGGTACACTGTCTATTACCCTATGCTGACTCAGAGTAAAGAGGACTTGGTTGTATGTAAGGATGGTAAATTTAGTAAGGTTCAGGTTAAAACAGCCACAACGGTTCCAACCAACACAGGAGATGCCAAGCAGGTTAGGCTAGGTGGATGCGGTAGGTCCGAATATAAGGATGGAGACTTTGACATTCTTGCGGTTGTGGTTGACGAAGATGTGCTTATTTTCACAt                                                                                                                                  <  1:355125/295‑1 (MQ=255)`  
                                                                                                                                                                          `aagCTACTCATGAAAGAAAGACACTTAACAGGTGCTGCCTCTGAAATGCTAGTAGCCTACAAAGTTACCAAAGCTGGGTACACTGTCTATTACCCTATGCTGACTCAGAGTAAAGAGGACTTGGTTGTATGTAAGGATGGTAAATTTAGTAAGGTTCAGGTTAAAACAGCCACAACGGTTCAAACCAACACAGGAGATGCCAAGCAGGTTAGGCTAGGTGGATGCGGTAGGTCCGAATATAAGGATGGAGACTTTGACATTCTTGCGGTTGTGGTTGACGAAGATGTGCTTATTTTCACAt                                                                                                                                  <  1:17040/299‑1 (MQ=255)`  
                                                                                                                                                                               `aCTCATGAACGAAAGACACTTAACAGGTGCTGCTTCTGAAATGCTAGTAGCCTACAAATTTACCAAAGCTGGGTACACTGTCTATTACCCTATGCTGACTCAGAGTAAAGAGGACTTTGTTGTATGTAAGGATGGTAAATTTAGTAAGGTTCAGGTTAAAACAGCCACAACGGTTCAAACCAACACAGGAGATGCCAAGCAGGTTAGGCTAGGTGGATGCGGTAGGTCCGAATATAAGGATGGAGACTTTGACATTCTTGCGGTTGTGGTTGACGAAGATGTGCTTATTTTCACATGGGAc                                                                                                                             >  1:333204/1‑301 (MQ=255)`  
                                                                                                                                                                                    `aaagagacggACACTTAACAGGTGCTGCTTCTGAAATGCTAGTAGCCTACAAATTTACCAAAGCTGGGTACACTGTCTATTACCCTATGCTGACTCAGAGTAAAGAGGACTTGGTTGTATGTAAGGATGGTAAATTTAGTAAGGTTCAGGTTAAAACAGCCACAACGGTTCAAACCAACACAGGAGATGCCAAGCAGGTTAGGCTAGGTGGATGCGGTAGGTCCGAATATAAGGATGGAGACTTTGACATTCTTGCGGTTGTGGTTGACGAAGATGTGCTTATTTTCACATGGGACGAAGt                                                                                                                        <  1:20564/292‑1 (MQ=255)`  
                                                                                                                                                                                     `gAACGAAAGACACTTAACAGGTGCTGCTTCTGAAATGCTAGTAGCCTACAAATTTACCAAAGCTGGGTACACTGTCTATTACCCTATGCTGACTCAGAGTAAAGAGGACTTGGTTGTATGTAAGGATGGTAAATTTAGTAAGGTTCAGGTTAAAACAGCCACAACGGTTCAAACCAACACAGGAGATGCCAAGCAGGTTAGGCTAGGTGGATGCGGTAGGTCCGAATATAAGGATGGAGACTTTGACATTCTTGCGGTTGTGGTTGACGAAGATGTGCTTATTTTAACATGGGACGAAGTa                                                                                                                       >  1:259111/1‑301 (MQ=255)`  
                                                                                                                                                                                        `cGAAAGACACTTAACAGGTGCTGCTTCTGAAATTCTAGTAGCCTACAAATTTACCAAAGCTGGGTACACTGTCTATTACCCTATGCTGACTCAGAGTAAAGAGGACTTGGTTGGATGTAAGGATGGTAAATTTAGTAAGGTTCAGGTTAAAACAGCCACAACGGTTCAAACCAACACAGGAGATGCCAAGCAGGTTAGGCTAGGTGGATGCGGTAGGTCCTAATATAAGGATGGAGACTTTGACATTCTTGCGGTTGTGGTTGACGAAGATGTGCTTATTTTCACATTGGACGAAGTaaaa                                                                                                                    <  1:652576/301‑1 (MQ=255)`  
                                                                                                                                                                                        `cGAAAGACACTTAACAGGTGCTGCTTCTGAAATGCTAGTAGCCTACAAATTTACCAAAGCTGGGTACACTGTCTATTACCCTATGCTGACTCAGAGTAAAGAGGACTTGGTTGTATGTAAGGATGGTAAATTTAGTAAGGTTCAGGTTAAAACAGCCACAACGGTTCAAACCCACACAGGAGATGCCAAGCAGGATAGGCTAGGTGGATGCGGTAGGTCCGAATATACGGATGGAGACTTTGACATTCTTGCGGTTGTGGTTGACGAAGATGTGCTTATTTTCACATGGGACGAAGTaaaa                                                                                                                    >  1:1740/1‑301 (MQ=255)`  
                                                                                                                                                                                          `acaGACACTTAACAGTTTCTGCATCTTAAATGCTAGTAGCCTACATATTTACCAAGTCTGGTTACACTGTCTATTACCCTATTCTGACTCAGAGTTAAGATGACTTGGTTGTATGTAAGGATTGTAAATTTAGTAAGGTTCAGGTTAAAACAGCCACAATGGTTCAAACCAACACAGGAGATGACAATCAGGTTAGGCTAGGTGGATGCGGTAGTTCCTAATATAAGGATGGAGACTTTGACATTCTTGCGGTTTTGGTTGACGTAGATGTGCTTATTTTCACATGGGACGAAGTAAAAgg                                                                                                                  <  1:53469/299‑1 (MQ=255)`  
                                                                                                                                                                                               `cacTTAACAGGTGCTGCTTCTGAAATGCTAGTAGCCTACAAATTTACCAAAGCTGGGTACACTGTCTATTACCCTATGCTGACTCAGAGTAAAGAGGACTTGGTTGTATGTAAGGATGGTAAATTTAGTAAGGTTCAGGTTAAAACAGCCACAACGGTTCAAACCAACACAGGAGATGCCAAGCAGGTTAGGCTAGGTGGATGCGGTAGGTCCGAATATAAGGATGGAGACTTTGACATTCTTGTGGTTGTGGTTGACGAAGATGTGCTTATTTTCACATGGGACGAAGTAAAAgctgtct                                                                                                             >  1:549926/1‑295 (MQ=255)`  
                                                                                                                                                                                                `acTTAACAGGTGCTGCTTCTGAAATGCTAGTAGCCTACAAATTTACCAAAGCTGGGTACACTGTCTATTACCCTATGCTGACTCAGAGTAAAGAGGACTTGGTTGTATGTAAGGATGGTAAATTTAGTAAGGTTCAGGTTAAAACAGCCACAACGGTTCAAACCAACACAGGAGATGCCAAGCAGGTTAGGCTAGGTGGATGCGGTAGGTCCGAATATAAGGATGGAGACTTTGACATTCTTGCGGTTGTGGTTGACGAAGATGTGCTTATTTTCACATGGGACGAAGTAAAAGGTAAGAc                                                                                                            <  1:464959/301‑1 (MQ=255)`  
                                                                                                                                                                                                 `cTTAACAGGTGCTGCTTCTGAAATGCTAGTAGCCTACAAATTTACCAAAGCTGGGTACACTGTCTATTACCCTATGCTGACTCAGCGTAAAGAGGACTTGGTTGTATGTAAGGATGGTAAATTTAGTAAGGTTCAGGTTAAAACAGCCACAACGGTTCAAACTAACACAGGAGATGCCAAGCAGGTTAGGCTAGGTGGATGCGGTAGGTCCGAATATAAGGATGGAGACTTTGACATTCTTGCGGTTGTGGTTGACGAAGATGTGCTTATTTTCACATGGCACGAAGTAAAAGGTAAGAcc                                                                                                           >  1:647713/1‑300 (MQ=255)`  
                                                                                                                                                                                                 `cTTAACAGGTGCTGCTTCTGAAATGCTAGTAGCCTACAAATTTACCAAAGCTGGGTACACTGTCTATTACCCTATGCTGACTCAGAGTAAAGAGGACTTGGTTGTATGTAAGGATGGTAAATTTAGTAAGGTTCAGGTTAAAACAGCCACAACGGTTCAAACCAACACAGGAGATGCCAAGCAGGTTAGGCTAGGTGGATGCGGTAGGTCCGAATATAAGGATGGAGACTTTGACATTCTTGCGGTTGTGGTTGACGAAGAGGTGCTTATTTTCACATGGGACGAAGTAAAAGGTAAGACa                                                                                                           >  1:95412/1‑301 (MQ=255)`  
                                                                                                                                                                                                       `aGGTGCTGCTTCTGAAATGCTAGTAGCCTACAAATTTACCAAAGCTGGGTACACTGTCTATTACCCTATGCTGACTCAGAGTAAAGAGGACTTGGTTGTATGTAAGGATGGTAAATTTAGTAAGGTTCAGGTTAAAACAGCCACAACGGTTCAAACCAACACAGGAGATGCCAAGCAGGTTAGGCTAGGTGGATGCGGTAGGTCAGAATATAAGGATGGAGACTTTGACATTCTTGCGGTTGTAGTTGACGAAGATGTGCTTATTTTCACATGGGACGAAGTAAAAGGTAAGACATCCAtg                                                                                                     <  1:104807/301‑1 (MQ=255)`  
                                                                                                                                                                                                                 `tCTGAAATGCTAGTAGCCTACAAATTTACCAAAGCTGGGTACACTGTCTATTACCCTATGCTGACTCAGAGTAAAGAGGACTTGGTTGTATGTAAGGATGGTAAATTTAGTAAGGTTCAGGTTAAAACAGCCACAACAGTTCAAACCAACACAGGAGATGCCAAGCAGGTTAGGCTAGGTGGATGCGGTAGGTCCGAATATAAGGATGGAGACTTTGACATTCTTGCGGTTGTGGTTGACGAAGATGTGCTTATTTTCACATGGGACGAAGTAAAAGGTAAGACATCCATGTGTGTAGGCa                                                                                           >  1:299034/1‑301 (MQ=255)`  
                                                                                                                                                                                                                          `cTAGTAGCCTACAAATTTACCAAAGCTGGGTACACTGTCTATTACCCTATGCTGACTCAGAGTAAAGAGGACTTTGTTGAATGTAAGGATGGTAACTTTAGTAAGGTTCAGGTTAAAACAGCCACAACGGTTCAAACCAACACAGGAGATGCCAAGCAGGTTAGGATAGGTGGATGCGGTAGGTACGAATATAAGGATGGAGACTTTGACATTCTTGCGGTTGTGGTTGACGCAGATGTGCTTATTTTCCCATGGGACGAAGAAAATGGTAAGACATCCATGTGTGTCGGCACGAGAcaca                                                                                  >  1:327234/1‑301 (MQ=255)`  
                                                                                                                                                                                                                             `gtagCCTACAAATTTACCAAAGCTGGGTACACTGTCTATTACCCTATGCTGACTCAGAGTAAAGAGGACTTGGTTGTATGTAAGGATGGTAAATTTAGTAAGGTTCAGGTTAAAACAGCCACAACGGTTCAAACCAACACAGGAGATGCCAAGCAGGTTAGGCTAGGTGGATGCGGTAGGTCCGAATATAAGGATGGAGACTTTGACATTCTTGCGGTTGTGGTTGACGAAGATGTGCTTATTTTCACATGGGACGAAGTAAAAGGTAAGACATCCATGTGTGTCGGCAAGAGAAACAAAg                                                                               <  1:162761/301‑1 (MQ=255)`  
                                                                                                                                                                                                                                 `ccTACAAATTTACCAAAGCTGGGTACACTGTCTATTACCCTATGCTGACTCAGAGTAAAGAGGACTTGGTTGTATGTAAGGATGGTAAATTTAGTAAGGTTCAGGTTAAAACAGCCACAACGGTTCAAACCAACACAGGAGATGCCAAGCAGGTTAGGCTAGGTGGATGCGGTAGGTCCGCATATAAGGATGGAGACTTTGACATTCTTGCGGTTGTGGTTGACGAAGATGTGCTTATTTTCACATGGGCCGCAGTAAAAGGTAAGTCATCCATGTGTGTCGGACAGAGAAACAGATGCAt                                                                           >  1:581408/1‑301 (MQ=255)`  
                                                                                                                                                                                                                                      `gtcggagatgtgtataagagacaggTCTATTACCCTATGCTGACTCAGAGTAAAGAGGACTTGGTTGTATGTAAGGATGGTAAATTTAGTAAGGTTCAGGTTAAAACAGCCAGAACGGTTCAAACCAACACAGGAGATGCCAAGCAGGTTAGGCTAGGTGGATGCGGTAGGTCCGAATATAAGGATGGAGACTTTGACATTCTTGCGGTTGTGGTTGACGAAGATGTGCTTATTTTCACATGGGACGAAGTAAAAGGTAAGACATCCATGTGTGTCGGCAAGAGAAACAAAGGCATAAAAc                                                                      <  1:383355/277‑1 (MQ=255)`  
                                                                                                                                                                                                                                      `aaaTGTACCAAAGCTGGGTACACTGTCTATTACCCTATGCTGACTCAGAGTAAAGAGGACTTGGTTGTATGTAAGGATGGTAAATTTAGTAAGGTTCAGGTTAAAACAGCCACAACGGTTCAAACCAACACAGGAGATGCCAAGCAGGTTAGGCTAGGTGGATGCGGTAGGTCCGAATATAAGGATGGAGACTTTGACATTCTTGCGGTTGTGGTTGACGAAGATGTGCTTATTTTCACATGGGACGAAGTAAAAGGTAAGACATTCATGTGTGTCGGCAAGAGAAACAAAGGCATAAAAc                                                                      <  1:289267/301‑1 (MQ=255)`  
                                                                                                                                                                                                                                        `aTTTACCAAAGCTTGGTACACTGTCTATTACCCTATGCTGACTCAGAGTAAAGAGGACTTGGTTGTATGTAAGGATGGTAAATTTAGTAAGGTTCAGGTTAAAACAGCCACAACGGTTCAAACCAACAAAGGAGATGCCAAGCAGGTTAGGCTAGGTGGATGCGGTAGGTCCGAATATAAGGATGGAGACTTTGACATTCTTGCGGTTGTGGTTGACGAAGATGTGCTTATTTTCACATGGGACGAAGTCAAAGGTAAGACATCCATGTGTGTCGACAAGAGAAACAAAGGCATAAAACtg                                                                    >  1:242861/1‑300 (MQ=255)`  
                                                                                                                                                                                                                                           `tACCAAAGCTGGGTACACTGTCTATTACCCTATGCTGACTCAGAGTAAAGAGGACTTGGTTGTATGTAAGGATGGTAAATTTAGTAAGGTTCAGGTTAAAACAGCCACAACGGTTCAAACCAACACAGGAGATGCCAAGCAGGTTAGGCTAGGTGGATGCGGTAGGTCCGAATATAAGGATGGAGACTTTGACATTCTTGCGGTTGTGGTTGACGAAGATGTGCTTATTTTCACATGGGACGAAGTAAAAGGTAAGACATCCATGTGTGTCGGCAAGAGAAACAAAGGctgtcgcttatac                                                                 >  1:316660/1‑289 (MQ=255)`  
                                                                                                                                                                                                                                            `aCCAAAGCTGGGTACACTGTCTATTACCCTATGCTGACTCAGAGTAAAGAGGACTTGGTTGTATGTAAGGATGGTAAATCTAGTAAGGTTCAGGTTAAAACAGCCACAACGGTTCAAACCAACACAGGAGATGCCAAGCAGGTTAGGCTAGGTGGATGCGGTAGGTCCGAATATAAGGATGGAGACTTTGACATTCTTGCGGTTGTGGTTGACGAAGATGTGCTTATTTTCACATGGGACGAAGTAAAAGGTAAGACATCCATGTGTGTCGGCAAGAGAAACAAAGGCATAAAACTATAgg                                                                <  1:281963/301‑1 (MQ=255)`  
                                                                                                                                                                                                                                             `acAAAGCTGGGTACACTGTCTATTACCCTATGCTGACTCAGAGTAAAGAGGACTTGGTTGAAGGTAAGGAGGGTAAATTTAGTGAGGTACAGGTTAAAACCGACACAACGGTTCAAACCAACACAGGAGAGGCCAAGCAGGTTAGGCTAGGTGGATGCGGTAGGTCCGAATATAAGGATGGAGACTTTGACATTCTTGCGGTTGTGGTTGACGAAGATGTGCTTATTTTCACATGGGACGAAGTAAAAGGTAAGACATCCATGTGTGTCGGCAAGAGAAACAAAGGCATAAAACTATAGga                                                               <  1:44877/300‑1 (MQ=255)`  
                                                                                                                                                                                                                                              `ggtgtataagagacaggtatATTACCCTATGCTGACTCAGAGTAAAGAGGAATTGGTTGTATGTAAGGATGGTAAATTTAGTAAGGTTCAGGTTAAAACAGCCACAACGGTTCAAACCAACACAGGAGATGCCAAGCAGGTTAGGCTAGGTGGATGCGGTAGGTCCGAATATAAGGATGGAGACTTTGACATTCTTGCGGTTGTGGTTGACGAAGATGTGCTTATTTTCACATGGGACGAAGTAAAAGGTAAGACATCCATGTGTGTCGGCAAGAGAAACAAAGGCATAAAACTATAGgag                                                              <  1:475761/282‑1 (MQ=255)`  
                                                                                                                                                                                                                                              `cAAAGCTGGGTACACTGTCTATTACCCTCTGCTGACGCAGAGTAAAGAGGACTTGGTTGTATGTAAGGATGGTAAATGTAGTAAGGTTCATGTTAAAACAGCCACAACGGTTCAAACCAACACAGGAGATGCCAAGCAGGTTAGGCTAGGTGGATGCGGTAGGTCCGAATATAAGGATGGAGACTTTGACATTCTTGCGGTTGTGGTTGACGAAGATGTGCTTATTTTCACATGGGACGAAGTAAAAGGTAAGACATCCATGTGTGTCGGCAAGAGAAACAAAGGCATAAAACTATAGgag                                                              <  1:390630/301‑1 (MQ=255)`  
                                                                                                                                                                                                                                                     `gggTACACTGTCTATTACCCTATGCTGACTCAGAGTAAAGAGGACTTGGTTGTATGTAAGGATGGTAAATTTAGTAAGGTTCAGGTTAAAACAGCCACAACGGTTCAAACCAACACAGGAGATGCCAAGCAGGTTAGGCTAGGTGGAGGCGGTAGGTCCGAATATAAGGATGGAGACTTTGAAATTCTAGCGGTTGTGGTTGACGAAGATGTGCTTATTTTCAAATGGGACGAAGTAAAAGGTAAGAAATCCATGTGTGTCGGCAAGAGAAAAAAAGGCATAAAACTAAAGGAGAActtct                                                       >  1:524724/1‑299 (MQ=255)`  
                                                                                                                                                                                                                                                       `aagagacagcTATTACCCTATGCTGACTCAGAGTAAAGAGGACTTGGTTGTATGTAAGGATGGTAAATTTAGTAAGGTTCAGGTTAAAACAGCCACAACGGTTCAAACCAACACAGGAGATGCCAAGCAGGTTAGGCTAGGTGGATGCGGTAGGTCCGAATATAAGGATGGAGACTTTGACATTCTTGCGGTTGTGGTTGACGAAGATGTGCTTATTTTCACATGGGACGAAGTAAAAGGTAAGACATCCATGTGTGTCGGCAAGAGAAACAAAGGCATAAAACTATAGGAGAAAttatta                                                     <  1:606006/292‑1 (MQ=255)`  
                                                                                                                                                                                                                                                          `atagacagaTTACCCTATGCTGACTCAGAGTAAAGAGGACTTGGTTGTATGTAAGGATGGTAAATTTAGTAAGGTTCAGTTTAAAACAGCCACAACGGTTCAAACCAACACAGGAGATGCCAAGCAGGTTAGGCTAGGTGGATGCGGTAGTTCCGAATATAAGGATGGAGACTTTGACATTCTTGCGGTTGTGGTTGACGAAGATGTGCTTATTTTCACATGGGACGAAGTAAAAGGTAAGACATCCATGTGTGTCGGCAAGAGAAACAAAGGCATAAAACTATAGGAGAAATTATTATgg                                                  <  1:152837/293‑1 (MQ=255)`  
                                                                                                                                                                                                                                                          `ataagagacagaCCCTATGCTGACTCAGAGTAAAGAGGACTTGGTTGTATGTAAGGATGGTAAATTTAGTAAGGTTCAGGTTAAAACAGCCACAACGGTTCAAACCAACACAGGAGATGCCAAGCAGGTTAGGCTAGGTGGATGCGGTAGGTCCGAATATAAGGATGGAGGCTTTGACATTCTTGCGGTTGTGGTTGACGAAGATGTGCTTATTTTCACATGGGACGAAGTAAAAGGTAAGACATCCATGTGTGTCGGCAAGAGAAACAAAGGCATAAAACTATAGGAGAAATTATTATgg                                                  <  1:336990/290‑1 (MQ=255)`  
                                                                                                                                                                                                                                                              `gTCTATTACCCTATGCTGACTCAGAGTAAAGAGGACTTGGTTGTATGTAAGGATGGTAAATTTAGTAAGGTTCAGGTTAAAACAGCCACAACGGTTCAAACCAACACAGGAGATGCCAAGCAGGTTAGGCTAGGTGGATGCGGTAGGTCCGAATATAAGGATGGAGACTTTGACGTTCTTGCGGTTGTGGTTGATGAAGATGTGCTTATTTTCACATGGGACGAAGTAAAAGGTAAGACATCCATGTGTGTCGGCAAGAGAAACAACGGCATAAAACTATAGGAGAAATTATTATGGCTAt                                              >  1:630245/1‑301 (MQ=255)`  
                                                                                                                                                                                                                                                              `gTCTATTACCCTATGCTGACTCAGAGTAAAGAGGACTTGGTTGTATGTAAGGATGGTAAATTTAGTAAGGTTCAGGTTAAAACAGCCACAACGGTTCAAACCAACACAGGAGATGCCAAGCAGGTTAGGCTAGGTGGATGCGGTAGGTCCGAATATAAGGATGGAGACTTTGACATTCTTGCGGTTGTGGTTGACGAAGATGTGCTTATTTTCACATGGGACGAAGTAAAAGGTAAGACATCCATGTGTGTCGGCAAGAGAAACAAAGGCATAAAACTATAGGAGAAATTATTATGGCTAt                                              >  1:420755/1‑301 (MQ=255)`  
                                                                                                                                                                                                                                                              `gTCTATTACCCTATGCTGACTCAGAGTAAAGAGGACTTGGTTGTATGTAAGGATGGTAAATTTAGTAAGGTTCAGGTTAAAACAGCCACAACGGTTCAAACCAACACAGGAGATGCCAAGCAGGTTAGGCTAGGTGGATGCGGTAGGTCCGAATATAAGGATGGAGACTTTGACATTCTTGCGGTTGTGGTTGACGAAGATGTGCTTATTTTCACATGGGACGAAGTAAAAGGTAAGACATCCATGTGTGTCGGCAAGAGAAACAAAGGCATAAAACTATAGGAGAAATTATTATGGCTAt                                              >  1:600773/1‑301 (MQ=255)`  
                                                                                                                                                                                                                                                              `gTCTATTACCCTATGCTGACTCAGAGTAAAGAGGACTTGGTTGTATGTAAGGATGGTAAATTTAGTAAGGTTCAGGTTAAAACAGCCACAACGGTTCAAACCAACACAGGAGATGCCAAGCAGGTTAGGCTAGGTGGATGCGGTAGGTCCGAATATAAGGATGGAGACTTTGACATTCTTGCGGTTGTGGTTGACGAAGATGTGCTTATTTTCACATGGGACGAAGTAAAAGGTAAGACATCCATGTGTGTCGGCAAGAGAAACAAAGGCATAAAACTATAGGAGAAATTATTATGGCTAt                                              >  1:561899/1‑301 (MQ=255)`  
                                                                                                                                                                                                                                                              `gTCTATTACCCTATGCTGACTCAGAGTAAAGAGGACTTGGTTGTATGTAAGGATGGTAAATTTAGTAAGGTTCAGGTTAAAACAGCCACAACGGTTCAAACCAACACAGGAGATGCCAAGCAGGTTAGGCTAGGTGGATGCGGTAGGTCCGAATATAAGGATGGAGACTTTGACATTCTTGCGGTTGTGGTTGACGAAGATGTGCTTATTTTCACATGGGACGAAGTAAAAGGTAAGACATCCATGTGTGTCGGCAAGAGAAACAAAGGCATAAAACTAGAGGAGAAATTATTATGGCTAt                                              >  1:610760/1‑301 (MQ=255)`  
                                                                                                                                                                                                                                                                `cTATTACCCTATGCTGACTCAGAGTAAAGAGGACTTGGTTGTATGTAAGGATGGTAAATTTAGTAAGGTTCAGGTTAAAACAGCCACAACGGTTCAAACCAACACAGGAGATGCCAAGCAGGTTAGGCTAGGTGGATGCGGTAGGTCCGAATATAAGGATGGAGACTTTGACATTCTTGCGGTTGTGGTTGACGAAGATGTGC‑TATTTTCACATGGGACGAAGTAAAAGGTAAGACATCCATGTGTGTCGGCAAGAGAAACAAAGGCATAAAACTATAGGAGAAATTATTATGGCTATGAc                                           >  1:191124/1‑301 (MQ=255)`  
                                                                                                                                                                                                                                                                `cTATTACCCTATGCTGACTCAGAGTAAAGAGGACTTGGTTGTATGTAAGGATGGTAAATTTAGTAAGGTTCAGGTTAAAACAGCCACAACGGTTCAAACCAACACAGGAGATGCCAAGCAGGTTAGGCTAGGTGGATGCGGTAGGTCAGAATATAAGGATGGAGACTTTGACATTCTTGCGGTTGTGGTTGACGAAGATGTGCTTATTTTCACATGGGACGAAGTAAAAGGTAAGACATCCATGTGTGTCGGCAAGAGAAACAAAGGAATAAAACTATAGGAGAAATTATTCTGGCTAGGa                                            >  1:573768/1‑301 (MQ=255)`  
                                                                                                                                                                                                                                                                 `acagaCCCTATGCTGACTCAGAGTAAAGAGGACTTGGTTGTATGTAAGGATGGTAAATTTAGTAAGGGTCAGGTTAAAACAGCCACAACGGTTCAAACCAACACAGGAGATGCCAAGCAGGTTAGTCTAGGTGGATGCGGTAGGTCCGAATATAAGGATGGAGACTTTGACATTCTTGCGGTTGTGGTTGACGAAGATGTGCTTATTTTCACATGGGACGAAGTAAAAGGTAAGACATCCATGTGTGTCGGCAAGAGAAACAAAGGCATAAAACTATAGGAGAAATTATTATGGCTATGAc                                           <  1:456450/297‑1 (MQ=255)`  
                                                                                                                                                                                                                                                                     `aCCCTATGCTGACTCAGAGTAAAGAGGACTTGGTTGTATGTAAGGATGGTAAATTTAGTAAGGTTCAGGTTAAAACAGCCACAACGGTTCAAACCAACACAGGAGATGCCAAGCAGGTTAGGCTAGGTGGATGCGGTAGGTCCGAATATAAGGATGGAGACTTTGACATTCTTGCGGTTGTGGTTGACGAAGATGTGCTTATTTTCACATGGGACGAAGTAAAAGGTAAGACATCCATGTGTGTCGGCAAGAGAAACAAAGGCATAAAACTATAGGAGAAATTATTATGGCTATGACAAAg                                       >  1:348956/1‑301 (MQ=255)`  
                                                                                                                                                                                                                                                                     `aCCCTATGCTGACTCAGAGTAAAGAGGACTTGGTTGTATGTAAGGATGGTAAATTTAGTAAGGTTCAGGTTAAAACAGCCACAACGGTTCAAACCAACACAGGAGATACCAAGCAGGTTAGGCTAGGGGGATGCGGTAGGTCCGAATATAAGGATGGAGACTTTGACATTCTTGCGGTTGTGGTTGACGAAGATGTGCTTATTTTCACATGGGACGAAGTAAAAGGTAAGAAATCCATGTGTGTCGGCAAGAGAAACAAAGGCATAAAACTATAGGAGAAATTATTATGGCTATGACaaaa                                       >  1:443057/1‑300 (MQ=255)`  
                                                                                                                                                                                                                                                                           `tGCTGACTCAGAGTAAAGAGGACTTGGTTGTATGTAAGGATGGTAAATTTAGTAAGGTTCAGGTTAAAACAGCCACAACGGTTCAAACCAACACAGGAGATGCCAAGCAGGTTAGGCTAGGTGGATGCGGTAGGTCCGAATATAAGGATGGAGACTTTGACATTCTTGCGGTTGTGGTTGACGAAGATGTGCTTATTTTCACATGGGACGAAGTAAAAGGTAAGACATCCATGTGTGTCGGCAAGAGAAACAAAGGCATAAAACTATAGGAGAAATTATTCTGTCtcttatacacatctcc                                 >  1:26736/1‑286 (MQ=255)`  
                                                                                                                                                                                                                                                                            `gCTGACTCAGAGTATAGAGGACTTGGTTGTATGTAAGGATGGTAAATTTAGTAAGGTTCAGGTTAAAACAGCCACAACGGTTCAAACCAACACAGGTGATGCCAAGCAGGTTAGGCTAGGTGGATGCGGTAGGTCTGAATATTAGGATGGAGACTTTGCCATTCTTGCGGTTGTGGTTGACGAAGATGTGCTTATTTTCACATGGGACGAAGTAAAAGGTAAGACATCCATGTGTGTATGCAAGAGAAACAAAGGCATAAGACTATAGGCGAAATTCTTATGTCTATGCCAAAGTAATTTa                                >  1:58485/1‑301 (MQ=255)`  
                                                                                                                                                                                                                                                                               `gACTCAGAGTAAAGAGGACTTGGTTGTATGTAAGGATGGTAAATTTAGTAAGGTTCAGGTTAAAACAGCCACAACGGTTCAAACCAACACAGGAGATGCCAAGCAGGTTAGGCTAGGTGGATGCGGTAGGTCCGAATATAAGGATTGAGACTTTGACATTCTTGCGGTTGTGGTTGACGAAGATGTGCTTATTTTCACATGAGACGAAGTAAAAGGTAAGACATCCATGTGTGTCGGCAAGAGAAACAAAGGCATAAAACTATAGGAGAAATTATTATGGCTCTgtctcttatacacatct                             >  1:211919/1‑285 (MQ=255)`  
                                                                                                                                                                                                                                                                                       `gTAAAGAGGACTTGGTTGTATGTAAGGATGGTAAATTTAGTAAGGTTCAGGTTAAAACAGCCACAACGGTTCAAACCAACACAGGAGATGCCAAGCAGGTTAGGCTAGGTGGATGCGGTAGGTCCGAATATAAGGATGGAGACTTTGACATTCTTGCGGTTGTGGTTGACGAAGATGTGCTTATTTTCACATGGGACGAAGTAAAAGGTAAGACATCCATGTGTGTCGGCAAGAGAAACAAAGGCATAAAACTATAGGAGAAATTATTATGGCTATGACAAAGAAATTTAAAGTGTCCTTc                     >  1:100102/1‑301 (MQ=255)`  
                                                                                                                                                                                                                                                                                       `gTAAAGAGGACTTGGTTGTATGTAAGGATGGTAAATTTAGTAAGGTTCAGGTTAAAACAGCCACAACGGTTCAAACCAACACAGGAGATGCCAAGCAGGTTAGGCTAGGTGGATGCGGTAGGTCCGAATATAAGGATGGAGACTTTGACATTCTTGCGGTTGTGGTTGACGAAGATGTGCTTATTTTCACATGGGACGAAGTAAAAGGTAAGACATCCATGTGTGTCGGCAAGAGAAACAAAGGCATAAAACTATAGGAGAAATTATTATGGCTATGACAAAGAAATTTAAAGTGTCCTTc                     <  1:90134/301‑1 (MQ=255)`  
                                                                                                                                                                                                                                                                                           `agagGACTTGGTTGTATGTAAGGATGGTAAATTTAGTAAGGTTCAGGTTAAAACAGCCACAACGGTTCAAACCAACACAGGAGATGCCAAGCAGGTTAGGCTAGGTGGATGCGGTAGGTCCGAATATAAGGATGGAGACTTTGACATTCTTGCGGTTGTGGTTGACGAAGATGTGCTTATTTTCACATGGGACGAAGTAAAAGGTAAGACATCCATGTGTGTCGGCAAGAGAAACAAAGGCATAAAACTATAGGAGAAATTATTATGGCTATGACAAAGAAATTTAAAGTGTCCTTCGACg                 <  1:593112/301‑1 (MQ=255)`  
                                                                                                                                                                                                                                                                                           `agagGAATTGGTTGTATGTAAGTATGATAAATTTAGTAACGTTCAGGTTAAAACCGTCTCAACGGTACAAACCAACACAGGATATGCCAAGTAGATTAGGCTAGGTGGAGGCGGTAGGTCCGAATATAAGGATGGAGACTTTGACATTCTTGCGGTTGTGGTTGACGAAGATGTGCTTATTTTCACATGAGACGAAGTAAAAGGTAAGACATCCATGAGTGTCGGCAAGAGAAACAAAGGCATAAAACTATAGGAGAAATTATTATGGCTATGACAAAGAAATTTAAAGTGTCCTTCGACg                 <  1:70066/301‑1 (MQ=255)`  
                                                                                                                                                                                                                                                                                              `ggACTTGGTTGTATGTAAGGATGGTAAATTTAGTAAGGTTCAGGTTAAAACAGCCACAACGGTTCAAACCAACACAGGAGATGCCAAGCAGGTTAGGCTAGGTGGATGCGGTAGGTCCGAATATAAGGATGGAGACTTTGACATTCTTGCGGTTGTGGTTGACGAAGATGTGCTTATTTTCACATGGGACGAAGTAAAAGGTAAGACATCCATGTGTGTCGGCAAGAGAAACAAAGGCATAAAACTATAGGAGAAATTATTATGGCTATGACAAAGAAATTTACAGTGTCCTTCGACGTTa              >  1:133697/1‑301 (MQ=255)`  
                                                                                                                                                                                                                                                                                                     `gTTGTATGTAAGGGTGGTAAATTTAGTAAGGTTCAGGTTAAAACCGCCACAACGGTTCAAACCAACACAGGAGATGCCAAGCAGGTTAGGCTAGGTGGATGCGGTAGGTCCGAATATAAGGATGGAGACTTTGACATTCTTGCGGTTGTGGTTGACGAAGATGTGCTTATTTTCACATGGGACGAAGTAAAAGGTAAGACATCCATGTTTGTCGGCAAGAGAAACAAAGGCATAAAACTATAGGAGAAATTATTATGGCTATGACAAAGAAATTTAAAGTGTCCTTCGACGTTACCGCaca       >  1:431736/1‑299 (MQ=255)`  
                                                                                                                                                                                                                                                                                                       `ggtatgtaAGGATGGTAAATTTAGTAAGGTTCAGGTTAAAACAGCCACAACGGTTCAAACCAACACAGGAGATGCCAAGCAGGTTAGGCTAGGTGGATGCGGTAGGTCCGAATATAAGGATGGAGACTTTGACATTCTTGCGGTTGTGGTTGACGAAGATGTGCTTATTTTCACATGGGACGAAGTAAAAGGTAAGACATCCATGTGTGTCGGCAAGAGAAACAAAGGCATAAAACTATAGGAGAAATTATTATGGCTATGACAAAGAAATTTAAAGTGTCCTTCGACGTTACCGCAAAGa     <  1:235048/300‑1 (MQ=255)`  
                                                                                                                                                                                                                                                                                                        `gtatgtaAGGATGGTAAATTTAGTAAGGTTCAGGTTAAAACAGCCACAACGGTTCAAACCAACACAGGAGATGCCAAGCAGGTTAGGCTAGGTGGATGCGGTAGGTCCGAATATAAGGATGGAGACTTTGACATTCTTGCGGTTGTGGTTGACGAAGATGTGCTTATTTTCACATGGGACGAAGTAAAAGGTAAGACATCCATGTGTGTCGGCAAGAGAAACAAAGGCATAAAACTATAGGAGAAATTATTATGGCTATGACAAAGAAATTTAAAGTGTCCTTCGACGTTACCGCaactgt    >  1:590042/1‑297 (MQ=255)`  
                                                                                                                                                                                                                                                                                                        `gtatgtaAGGATGGTAAATTTAGTAAGGTTCAGGTTAAAACAGCCACAACGGTTCAAACCAACACAGGAGATGCCAAGCAGGTTAGGCTAGGTGGATGCGGTAGGTCCGAATATAAGGATGGAGACTTTGACATTCTTGCGGTTGTGGTTGACGAAGATGTGCTTATTTTCACATGGGACGAAGTAAAAGGTAAGACATCCATGTGTGTCGGCAAGAGAAACAAAGGCATAAAACTATAGGAGAAATTATTATGGCTATGACAAAGAAATTTAAAGTGTCCTTCGACGTTACCGCAAAGAt    >  1:437371/1‑301 (MQ=255)`  
                                                                                                                                                                                                                                                                                                        `gcatgtaAGGATGGTAAATTTAGTAAGGTTCAGGTTAAAACAGCCACAACGGTTCAAACCAACACAGGAGATGCCAAGCAGGTTAGGCTAGGTGGATGCGGTAGGTCCGAATATAAGGATGGAGACTTTGACATTCTTGCGGTTGTGGTTGACGAAGAGGTGCTTATTTCCACATGGGACGAAGTAAAGGGTAAGAAATCCATGTGTGTCGGCAGGAGAAACAAAGGAAGAAAACTATAGGAGAAATTATTATGTCTATGACAAAGAAATTAAAGGTGTCCTTCGACGTTACCGCaacaat    >  1:409705/3‑297 (MQ=255)`  
                                                                                                                                                                                                                                                                                                          `atgtaAGGCTGGTAAATTTAGTAAGGTTCAGGTTAAAACAGCCACAACGGTTCAAACCAACACAGGAGATGCCAAGCAGGTTAGGCTAGGTGGATGCGGTAGGTCCGAATATAAGGATGGAGACTTTGACATTCTTGCGGTTGTGGTTGACGAAGATGTGCTTATTTTCACATGGGACGAAGTAAAAGGTAAGACATCCATGTGTGTCGGCAAGAGAAACAAAGGCATAAAACTATAGGAGAAATTATTATGGCTATGACAAAGAAATTTAAAgtgctgtctcttatacacatctccgagc  >  1:341593/1‑276 (MQ=255)`  
                                                                                                                                                                                                                                                                                                          `atgtaAGGCTGGTAAATTTAGTAAGGTTCAGGTTAAAACAGCCACAACGGTTCAAACCAACACAGGAGATGCCAAGCAGGTTAGGCTAGATGGATGCGGTAGGTCCGAATATAAGGATGGAGACTTTGACATTCTTGCGGTTGTGGTTGACGAAGCTGTGCTTATTTTCACATGGGACGAAGTAAAAGGTAAGACATCCATGTGTGTCGGCAAGAGAAACAAAGGCATAAAACTATAGGAGAAATTATTATGGCTATGACAAAGAAATTTAAAGTGTCCTTCGACGTTACCGCAAAGATgg  >  1:37058/1‑300 (MQ=255)`  
                                                                                                                                                                                                                                                                                                          `atgtaAGGATGGTAAATTTAGTAAGGTTCGGGTTAAAACAGCCACAACGGTTCAAACCAACACAGGAGATGCCAAGCAGGTTAGGCTAGGTGGATGCGGTAGGTCCGAATATAAGGATGGAGACTTTGACATTCTTGCGGTTGTGGTTGACGAAGATGTGCTTATTTTCACATGGGACGAAGTAAAAGGTAAGACATCCATGTGTGTCGGCAAGAGAAACAAAGGCATAAAACTATAGGAGAAATTATTATGGCTATGACAAAGAAATTTAAAGTGTCCTTCGACGTTACCGCAAAGATgt  >  1:556689/1‑301 (MQ=255)`  
                                                                                                                                                                                                                                                                                                          `atgtaAGGATGGTAAATTTAGTAAGGTTCAGGTTAAAACAGCCACAACGGTTCAAACCAACACAGGAGATGCCAAGCAGGTTAGGCTAGGTGGATGCGGTAGGTCCGAATATAAGGATGGAGACTTTGACATTCTTTCGGTTGTGGTTGACGAAGATGTGCTTATTTTCACATGGGACGAAGTAAAAGGTAAGACATCCATGTGTGTCGGCAAGAGAAACAAAGGCATAAAACTATAGGAGAAATTATTATGGCTATGACAAAGAAATTTAAAGTGTCCTTCGACGTTACCGCAAAGATgt  >  1:184124/1‑301 (MQ=255)`  

                                                                                                                                                                                                                                                                                                            `|`                                                                                                                                                                                                                                                                                                            
`GGGTACATGATGAAATCCAAGTAGGCTGCCGTACCGAAGAGATTGCTCAGGTGGTCATTGAGACCGCACAAGAAGCGATGCGCTGGGTTGGAGACCACTGGAACTTCCGGTGTCTTCTGGATACCGAAGGTAAGATGGGTCCTAATTGGGCGATTTGCCACTGATACAGGAGGCTACTCATGAACGAAAGACACTTAACAGGTGCTGCTTCTGAAATGCTAGTAGCCTACAAATTTACCAAAGCTGGGTACACTGTCTATTACCCTATGCTGACTCAGAGTAAAGAGGACTTGGTTGTATGTAAGGATGGTAAATTTAGTAAGGTTCAGGTTAAAACAGCCACAACGGTTCAAACCAACACAGGAGATGCCAAGCAGGTTAGGCTAGGTGGATGCGGTAGGTCCGAATATAAGGATGGAGACTTTGACATTCTTGCGGTTGTGGTTGACGAAGATGTGCTTATTTTCACATGGGACGAAGTAAAAGGTAAGACATCCATGTGTGTCGGCAAGAGAAACAAAGGCATAAAACTATAGGAGAAATTATTATGGCTATGACAAAGAAATTTAAAGTGTCCTTCGACGTTACCGCAAAGATGT  >  NC_001604.1/16304‑16902`

  

Alignment Legend

Aligned base mismatch/match (shaded by quality score): `ATCG/ATCG` `< 3 ≤` `ATCG/ATCG` `< 11 ≤` `ATCG/ATCG` `< 23 ≤` `ATCG/ATCG` `< 34 ≤` `ATCG/ATCG` `< 38 ≤` `ATCG/ATCG`

Unaligned base: `atcg`    Masked matching base: `atcg`    Alignment gap: `‑`    Deleted base: `‑`

Reads not counted as support for junction

read\_name Not counted due to insufficient overlap past the breakpoint.

read\_name Not counted due to not crossing MOB target site duplication.
